# Rapid isolation and enrichment of extracellular vesicle preparations using anion exchange chromatography

**DOI:** 10.1038/s41598-018-24163-y

**Published:** 2018-04-10

**Authors:** Nikki Heath, Lois Grant, Taiana Maia De Oliveira, Rachel Rowlinson, Xabier Osteikoetxea, Niek Dekker, Ross Overman

**Affiliations:** 1 Discovery Sciences, IMED Biotech Unit, AstraZeneca, Alderley Park, UK; 2 Discovery Sciences, IMED Biotech Unit, AstraZeneca, Cambridge, UK; 3 Discovery Sciences, IMED Biotech Unit, AstraZeneca, Gothenburg, Sweden

## Abstract

Extracellular vesicles (EVs) have important roles in physiology, pathology, and more recently have been identified as efficient carriers of therapeutic cargoes. For efficient study of EVs, a single-step, rapid and scalable isolation strategy is necessary. Chromatography techniques are widely used for isolation of biological material for clinical applications and as EVs have a net negative charge, anion exchange chromatography (AIEX) is a strong candidate for column based EV isolation. We isolated EVs by AIEX and compared them to EVs isolated by ultracentrifugation (UC) and tangential flow filtration (TFF). EVs isolated by AIEX had comparable yield, EV marker presence, size and morphology to those isolated by UC and had decreased protein and debris contamination as compared to TFF purified EVs. An improved AIEX protocol allowing for higher flow rates and step elution isolated 2.4*10^11^ EVs from 1 litre of cell culture supernatant within 3 hours and removed multiple contaminating proteins. Importantly AIEX isolated EVs from different cell lines including HEK293T, H1299, HCT116 and Expi293F cells. The AIEX protocol described here can be used to isolate and enrich intact EVs in a rapid and scalable manner and shows great promise for further use in the field for both research and clinical purposes.

## Introduction

Extracellular vesicles (EVs) are a family of membrane enclosed vesicles involved in cell-cell communication. The most well-known and studied members of the extracellular vesicle family are multivesicular body derived exosomes (<100 nm) and plasma membrane derived microvesicles (<1 µm)^1^. Due to their similar physical characteristics, the two vesicle populations can be difficult to separate effectively and so collectively they are termed EVs^2^. EVs contain lipids, proteins and nucleic acids, all of which can be delivered in a functional capacity to recipient cells leading to a change in the receiving cell phenotype. This process can either be beneficial in normal physiology, for example in immune system communication^3^ and neuroprotection^4^, as well as be detrimental in pathological diseases such as cancer^5^, neurodegeneration^6^ and atherosclerosis^7,8^.

It is the ability of EVs to transfer or deliver messages from one cell to another that makes them promising candidates to exploit for therapeutic purposes. There are many advantages of using isolated EVs as therapeutic delivery vehicles to reverse a pathological phenotype in diseased tissues, as reviewed by Jiang *et al*. in 2017^9^. For example, the administration of autologous EVs would be unlikely to activate the host immune response meaning repeated administration would be possible^10^ (contrary to viral delivery vehicles). EVs could also be engineered to target delivery of their contents only to diseased cells resulting in fewer side effects and fewer off target effects, improving efficacy and therapeutic margin of the chosen cargo^9,11^. The apparent low toxicity and immunogenicity of cell line-derived EVs *in vivo* suggests that large scale and efficient engineering of cell line-derived cargo carrying EVs could eventually be achieved and stored in potent doses for administration^12^.

The range of different therapeutic cargoes that have been demonstrated to be successfully delivered by EVs is expanding rapidly. Nucleic acids are the most common exogenous payload investigated to date with numerous studies detailed in the literature. EVs have been engineered to carry siRNA to mutant kRas^G12D^ and administration of these exosomes to pancreatic cancer mouse models driven by said mutation increased the overall survival of mice^13^. In a separate study, the targeted delivery of exosomes containing Let-7a micro-RNA to epidermal growth factor receptor positive breast cancer tumours was achieved, with a resultant inhibition of tumour growth observed^11^. In terms of small molecules, the delivery of EV encapsulated doxorubicin to αv integrin expressing breast cancer cells was found to inhibit tumour growth^14^. Even large proteins such as catalase, have been successfully delivered to mouse models of Parkinson’s disease using loaded EVs, giving rise to neuroprotective effects^15^. Most of the work around EV therapeutics so far has been undertaken in animal models; however, there are some cases where EVs have been taken into clinical trials. Autologous EVs derived from patients’ dendritic cells that were subsequently loaded with natural class II peptide or tumour associated antigen peptides, have been dosed back to patients and resulted, in some cases, in stabilisation of melanoma disease and non-small cell lung carcinoma respectively^16,17^.

A key caveat to the clinical exploitation of EVs is that they are difficult to isolate in a timely and cost-effective fashion with the required purity and at a suitable scale. Therefore, a methodology that will allow the rapid isolation of multiple doses of pure EVs from autologous or engineered cells would greatly facilitate such applications.

A commonly used technique for EV isolation is differential ultracentrifugation (UC). Cell culture supernatant is subjected to multiple centrifugation steps at increasing speeds to sequentially deplete the media of cells and debris and ultimately pelleting the desired microvesicle and exosome fractions^18^ (see Supplementary Fig. 1A). Although effective at pelleting EVs, UC cannot be used to completely separate EV populations and can also co-isolate contaminating protein aggregates^19^. UC can be used in isolation or in conjunction with density gradients and sucrose cushions to further separate EV subpopulations based on their density and to aid in the removal of contaminating proteins respectively^18^. This however is time consuming and difficult to scale up for EV production in a clinical laboratory environment with the required reproducibility. Alternatively, some recent clinical studies have employed tangential-flow filtration (TFF) for EV isolation^16,20^. In this technique, the conditioned cell culture supernatant is both concentrated and filtered at the same time using a peristaltic system with a high molecular weight cut off membrane filter (see Supplementary Fig. 1B). This technique is readily scalable but often requires a second process such as further filtration, size-exclusion chromatography, or a sucrose/deuterium density cushion to achieve the optimal purity which consequently increases processing time and can reduce overall EV yield^20–22^.

An alternative technique that is increasingly being applied to isolate EVs is the use of precipitants such as polyethylene glycol combined with low speed centrifugation to pellet EVs for subsequent processing^23^. Immuno-affinity capture has also been shown to be suitable to specifically isolate CD63/CD9/CD81 positive EVs^24^ however if one needs to isolate EVs at large scale, the EVs would need to be pre-concentrated with another method. In addition, the binding is not easily reversible, making applications beyond Western blotting or flow cytometry studies more challenging. Affinity capture methods can however be adapted to be reversible, for example using Tim4 peptide, the interaction of which is Ca^2+^ dependent, such that upon addition of a Ca^2+^ chelator the EVs dissociate from the beads^25^. One notable study used immuno-affinity capture beads to bind EpCam positive EVs which can then be eluted using a low pH buffer^26^.

More recently, size exclusion chromatography has gained a lot of attention in the EV isolation field as an efficient single-step method for EV isolation from small quantities of biological fluids^22^. For large scale EV production this method may be substantially more time consuming as one first must concentrate the EVs using a method such as UC or TFF before applying them to the chromatography matrix; in addition, the eluate from the column may require additional concentration steps. Such a protracted multi-step process could result in the reduction of EV yield and quality whilst increasing the likelihood of introducing unwanted environmental contaminants. Bind elute size exclusion chromatography can be used to avoid sample dilution upon elution. The column matrix traps and removes smaller contaminating proteins (<700 kDa) and allows EVs to pass directly through. However, a pre-concentration step by TFF is still necessary before application to the column^27^. For clinical applications that require large scale EV production the desired isolation protocol would be a single-step, automatable and reproducible method that isolates EVs directly from large volumes of biological fluid or cell culture conditioned media with the required high levels of purity. Here we propose anion exchange (AIEX) chromatography as a suitable candidate that could fulfil these stringent criteria.

AIEX chromatography (outlined in Supplementary Fig. 1C) exploits the net negative charge found on the EV surface^28^ to bind the particles to a positively charged chromatographic matrix; the bound EVs can then be eluted by increasing the ionic strength of the surrounding mobile phase. AIEX using solid-phase monolithic columns has been used to isolate virus-like particles (VLPs) from both clarified yeast lysates^29^, and CHO cell culture supernatants, and separate them from contaminating particles and DNA^30^. Whilst mesenchymal stem cell-derived EVs have been isolated using bead based anion exchange chromatography^31^, there have been no comprehensive studies published that scrutinise the utility of monolithic AIEX as a single-step EV isolation technique alongside the more common techniques of UC and TFF.

In this study, we demonstrate the isolation of HEK293T cell-derived EVs by AIEX and compare the yield, quality and purity of these preparations to those isolated using TFF and UC single-step protocols. We demonstrate that AIEX isolates EVs of a similar yield, purity and size distribution as UC in a single-step process that is easily scalable, whilst in contrast; EVs isolated by TFF were found to be contaminated with debris and protein. Subsequent optimisation of the AIEX protocol by increasing flow rates and introducing step gradients not only increased the speed of EV isolation but also the purity by enabling the removal of contaminating proteins. We believe that the simple AIEX method outlined herein will prove to be a useful technique for the rapid isolation of high quality EV preparations for research scale activities, *in vivo* studies and large-scale clinical applications.

## Results

### Monolithic AIEX efficiently isolates EVs from conditioned cell supernatants

**Figure 1:**
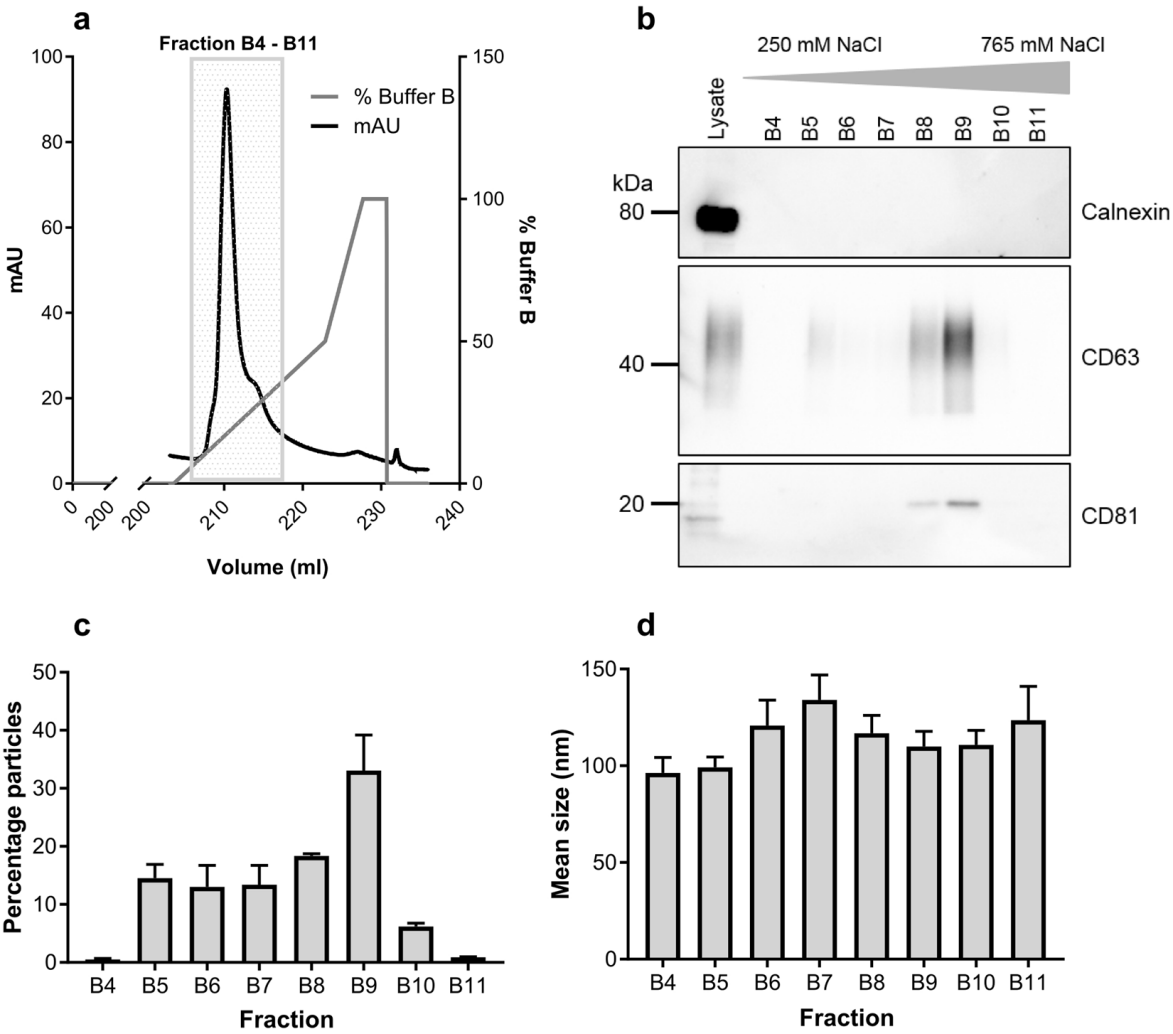
AIEX chromatography can be used to isolate EVs. (**a**) Conditioned media from HEK293T cells was applied to a 1 ml monolithic anion exchange column at 2 ml/minute. Bound particles were then eluted using a linear salt gradient up to 1 M NaCl using buffer B (50 mM HEPES, 2 M NaCl) and eluted proteins detected by UV absorbance at 280 nm (mAU - milli absorbance units) as shown in the representative UV chromatogram. (**b**) The main products collected were eluted between 250 mM and 765 mM NaCl (fractions B4–B11), and these contained EV markers in Western blot analysis. Full length Western blots can be seen in Supplementary Fig. 3. (**c**,**d**) NTA shows that the majority of fractions B4–B11 contained particles of an EV size n = 3. Error bars ± SEM.

As the starting point for this study we chose to adapt a protocol used by Steppert *et al*.^30^ for the isolation of VLPs to determine the efficiency of AIEX for the isolation of EVs from HEK293T cell-conditioned supernatants. Conditioned media from an average of 6.4*10^8^ cells (in 210 ml media) was clarified using a 10 minute 300 g spin followed by 0.2 µm filtration, and was applied to a 1 ml monolithic AIEX column at 2 ml/minute. Upon completion of the binding phase, bound materials were eluted using a linear gradient of 100 mM to 1 M NaCl over 20 column volumes (See Supplementary Fig. 1 and Methods for further details). An increase in the UV absorbance profile for the elution phase was detected between 250–765 mM NaCl (Fig. 1a and Supplementary Fig. 2 shows the whole chromatogram), this increase corresponded to the presence of EV markers CD63 and CD81, which were detected in these fractions by Western blot (Fig. 1b – see Supplementary Fig. 3 for full Western blot images). Importantly, calnexin, a common contaminant was not detectable in the EV positive fractions. Furthermore, Nanoparticle tracking analysis (NTA) showed the presence of EV sized vesicles (mean minimum: 96 nm, mean maximum: 134 nm) in the majority of these fractions (Fig. 1c,d).

### Comparing AIEX, UC and TFF isolated EVs

With the goal of rapid, single-step and scalable EV isolation in mind, EVs isolated by our AIEX protocol were compared to those isolated by UC and TFF. The schematic diagram in Supplementary Fig. 1 outlines the protocols that were followed for EV isolation by UC (A), TFF (B) and AIEX (C).

**Figure 2:**
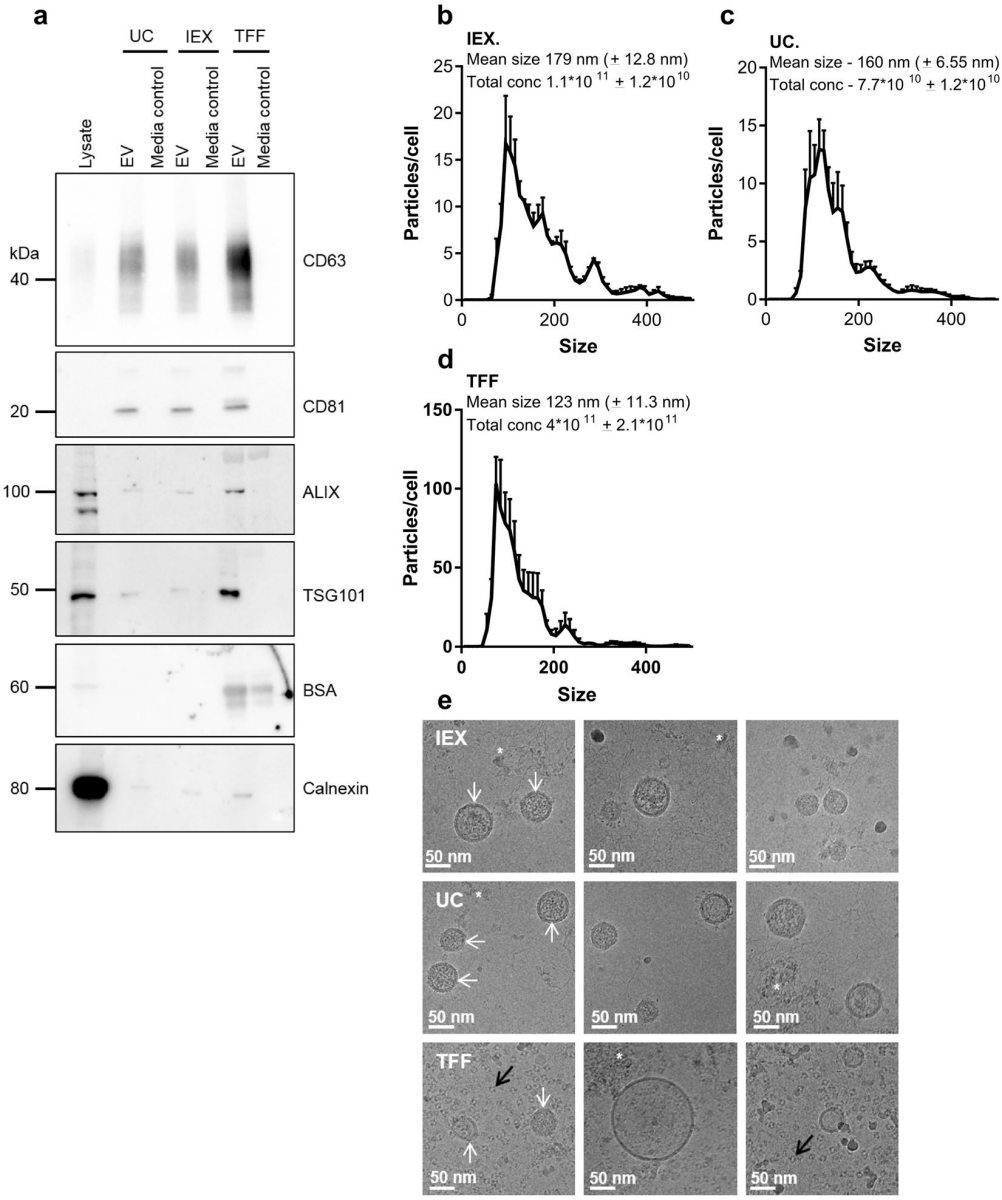
AIEX-isolated EVs have comparable EV marker content and size distribution profiles to those isolated by UC but not those isolated by TFF. EVs were isolated from HEK293T conditioned media by AIEX, UC and TFF. (**a**) Shows Western blot for EV markers (CD63, CD81, ALIX, TSG101) and potential preparation contaminants (BSA, Calnexin) in cell lysates and EVs isolated from clarified media and a media-only control (10% EV depleted FBS containing media that had not been exposed to cells) by UC, AIEX and TFF n = 3. Full length Western blots can be found in Supplementary Fig. 4. (**b**–**d**) Nanoparticle tracking analysis data showed the size distribution (data shown for 1–499 nM only, with a 10 nm bin width), average concentration and average size of EVs isolated by each technique n = 3. Error bars ± SEM. (**e**) Cryo-electron microscopy images of EVs after isolation by AIEX, UC and TFF. White arrows indicate EVs. Black arrows indicate lipid droplets in TFF sample. Asterisks indicate areas showing protein contamination n = 3. Scale bar = 50 nm.

Western blot analysis (Fig. 2a, and full blots in Supplementary Fig. 4) demonstrated that EV preparations isolated by UC, TFF and AIEX each contain the common EV markers CD63, CD81, ALIX and TSG101. Media-only controls were used to check for contaminating bovine EVs and media-derived proteins. Neither AIEX nor UC isolated media-only controls contained any evidence of EV markers or BSA. However, TFF isolated EVs and media-only controls were contaminated with BSA, a common contaminant of EV preparations. Upon blot over-exposure, a weak signal for calnexin could be detected in EV preparations. Nanoparticle tracking analysis showed that AIEX and UC isolated EVs had a similar yield and size distribution (Fig. 2b,c). Whereas EVs isolated by TFF were between 3.6 and 5 times more concentrated than AIEX and UC isolated EVs respectively, although this was not significant due to variability between experiments (Fig. 2d). It is worth noting that the concentration of EVs isolated by AIEX and UC was more reproducible between experiments than EVs isolated by TFF, with a much smaller standard error of the mean (SEM) observed.

Cryo-electron microscope imaging showed that EVs isolated by all three techniques were intact and of the expected morphology (Fig. 2e). There was some protein contamination observed in the UC and AIEX isolated EVs but very little compared to that observed in TFF preparations. Upon further observation of TFF samples there were small lipid- like droplets visible as well as protein. This indicated that TFF was a less suitable technique for scalable single-step EV isolation compared with UC and AIEX due to the higher levels of impurities.

### Optimised AIEX protocol improves speed of isolation and purity of EV preparations

**Figure 3:**
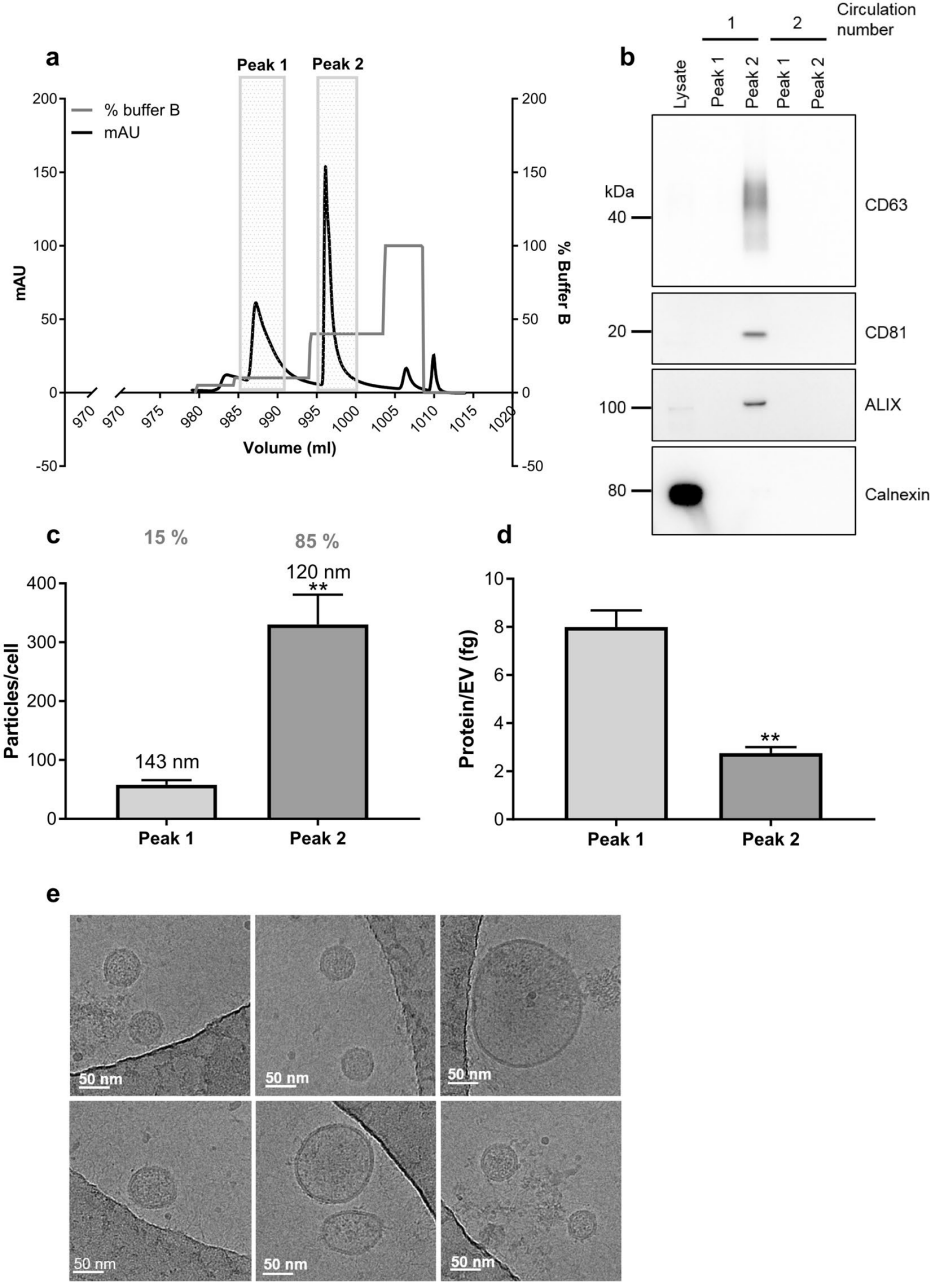
AIEX protocol modification makes EV isolation faster, more scalable and removes contaminating proteins. (**a**) Chromatogram shows UV absorbance at 280 nm (mAu) during the elution of EVs from AIEX column using a 335 mM (10% buffer B) and 890 mM (40% buffer B) step elution protocol. n = 3. (**b**) Western blot of EVs isolated from peak 1 and peak 2 after the first and second time conditioned media was passed through the AIEX column using the modified AIEX protocol. Samples were blotted for EV markers CD63, CD81 and ALIX as well as for common contaminant calnexin n = 3. Full length Western blots can be found in Supplementary Fig. 6. (**c**) Nanoparticle tracking analysis allowed calculation of average particle concentration and mean EV size (annotated on graph) isolated from peak 1 and 2. Also annotated is percentage of particles that were found in each peak n = 3. Error bars ± SEM. Unpaired two tailed t-test, **p < 0.01. (**d**) The protein concentration of each peak was taken using Nanodrop, and the particle concentrations as analysed by NTA were used to calculate protein/particle concentration n = 3. Error bars ± SEM. Unpaired two tailed t-test, **p < 0.01. (**e**) Cryo-electron microscopy of EVs eluted with 890 mM NaCl (40% Buffer B) in peak 2 n = 3. Scale bar = 50 nm.

Next, we were interested to investigate whether we would be able to optimise the AIEX protocol further to remove contaminating proteins as well as reduce the overall processing time. In previous studies used to isolate VLPs, a step gradient was employed to separate VLPs from other vesicular contaminants^30^. Therefore, we used a step gradient approach of 335 mM NaCl over 10 CV and 890 mM NaCl over 10 CV to separate contaminating proteins away from the EVs. An elevated flow rate of 10 ml/minute was also used to increase the speed of EV collection from 1 litre of HEK293T-conditioned media. This modified AIEX protocol resulted in the elution of two defined peaks, the fractions from which were then pooled, buffer exchanged and concentrated for analysis (Fig. 3a, Supplementary Fig. 5 shows the whole chromatogram). Western blotting revealed that EV markers CD63, CD81 and ALIX were predominantly present in peak 2 and calnexin was largely undetectable (Fig. 3b, see Supplementary Fig. 6 for full blots). Interestingly when the flow-through was re-circulated over the column, only very small peaks were observed upon subsequent elution and no EV markers (aside from a weak CD63 signal upon overexposure) were detected by Western blot compared to the initial peak 2 EV marker signal (Fig. 3b). Overall this data suggests that the majority of EVs released by 7.8*10^8^ cells into 1 litre of media during the 48-hour conditioning period were isolated by the 1 ml AIEX column in the first pass. Furthermore, NTA showed that peak 2 contained the majority of EVs (2.6*10^11^) with an average size of 119 nm, whereas peak 1 contained approximately 6 times fewer particles (4.4*10^10^) (Fig. 3c). Interestingly the protein concentration per particle was higher in peak 1 than that in peak 2 (Fig. 3d). Altogether this data shows that the modified protocol successfully enriches for EVs in peak 2 (890 mM NaCl) and removes contaminating proteins from the EVs into peak 1 (335 mM NaCl). Finally, cryo-electron microscope analysis showed that the EVs isolated by this optimised protocol were intact and had low levels of protein contamination (Fig. 3e).

To confirm that the modified AIEX protocol enriches for EVs in peak 2, peptide mapping of proteins isolated from both peaks was performed. The proteins significantly enriched in each peak were used to analyse EV marker content. 27 of the top 50 exosome markers (as identified by exocarta database^32^), were significantly enriched in peak 2 compared to 6 that were significantly enriched in peak 1. Interestingly one of the proteins found in peak 1 was albumin, a common contaminant of EV preparations, indicative that AIEX removes the protein contaminant efficiently (Table 1). The data was analysed using web based gene set analysis toolkit (WebGestalt)^33^ which collates the identified proteins into groups where they have common functional pathways and cellular compartments. The most common pathway proteins identified in peak 1 were involved in was the ribosomal pathway and the most common cellular compartment in which the proteins resided were in macromolecular complexes. Whereas the most common pathway to which proteins of peak 2 were attributed was endocytosis and the majority of proteins identified were of membrane origin. This analysis supports that plasma membrane-derived/MVB-derived EVs were being isolated in Peak 2, whereas contaminating proteins such as ribosomal proteins and BSA were being eluted earlier than EVs in peak 1 (see Supplementary Fig. 7). All proteins identified as enriched in peak 1 and peak 2 can be found in the Supplementary excel Files 1 and 2.

**Table 1:** Peptide mapping shows that the AIEX step elution protocol enriched for EVs in peak 2.

Top 50 Exosome markers from exocarta			
Gene Symbol	Enriched in peak:	Gene Symbol	Enriched in peak:
CD9	2	VCP	
HSPA8	2	TPI1	2
PDCD6IP	2	PPIA	2
GAPDH	1	MSN	1
ACTB		CFL1	
ANXA2	2	PRDX1	2
CD63	2	PFN1	2
SDCBP	2	RAP1B	2
ENO1		ITGB1	2
HSP90AA1		HSPA5	
TSG101		SLC3A2	1
PKM	2	HIST1H4A	1
LDHA	2	GNB2	2
EEF1A1	2	ATP1A1	2
YWHAZ	2	YWHAQ	2
PGK1	2	FLOT1	
EEF2		FLNA	
ALDOA	2	CLIC1	
HSP90AB1	1	CDC42	2
ANXA5	2	CCT2	1
FASN		A2M	
YWHAE	2	YWHAG	2
CLTC		TUBA1B	
CD81	2	RAC1	2
ALB	1	LGALS3BP	

Peak 1 and peak 2 were lysed in 6 M urea and subjected to peptide mapping. The top 50 proteins reported to be found in exosomes on exocarta^32^ were then used to determine whether the majority of EV proteins resided in peak 1 or in peak 2 n = 3. ANOVA calculated by Waters progenesis software. Proteins with a p value of 0.05 or less were included in this subsequent analysis.

EV preparation contamination with FBS derived proteins such as BSA and apolipoproteins, the protein constituents of lipoproteins, such as ApoB and ApoA1 is an increasingly recognised problem^34^. Apolipoproteins have been shown to be purified by AIEX from plasma^35^, therefore we sought to determine whether FBS derived apolipoproteins were present in EV preparations isolated by AIEX. 7.5 µl of Peak 1 and peak 2 which were in a total final volume of 100 µl, and 7.5 µl of unconcentrated conditioned media before AIEX, and post AIEX flow-through (total volume of 1000 ml) were Western blotted for BSA and ApoB and ApoA1.

Western blots in Supplementary Fig. 8 show that EV marker ALIX was enriched in peak 2 and was not present in the clarified conditioned media pre/post AIEX. ApoB was shown to be present in equal amounts in peak 1, peak 2 and in clarified conditioned media pre- and post-AIEX, although at very low levels compared to 10 µg HEPG2 lysate positive control. This indicates that AIEX does not enrich for ApoB with EVs. ApoA1 was found to be undetectable in peak 1 and peak 2 further supporting the fact that the AIEX method did not enrich for apolipoproteins with the EVs (see Supplementary Fig. 9). BSA was in very high abundance in the clarified conditioned media pre/post AIEX, with low amounts detected in peak 1 and 2. Corresponding to mass spectrometry data, BSA was commonly enriched in Peak 1 separated from the EVs in peak 2 (see Supplementary Fig. 9). Overall the data suggests that EVs were enriched in peak 2 and that the AIEX method does not enrich for apolipoproteins or BSA within the same EV fraction.

### EVs can be isolated from different cell types using AIEX

**Figure 4:**
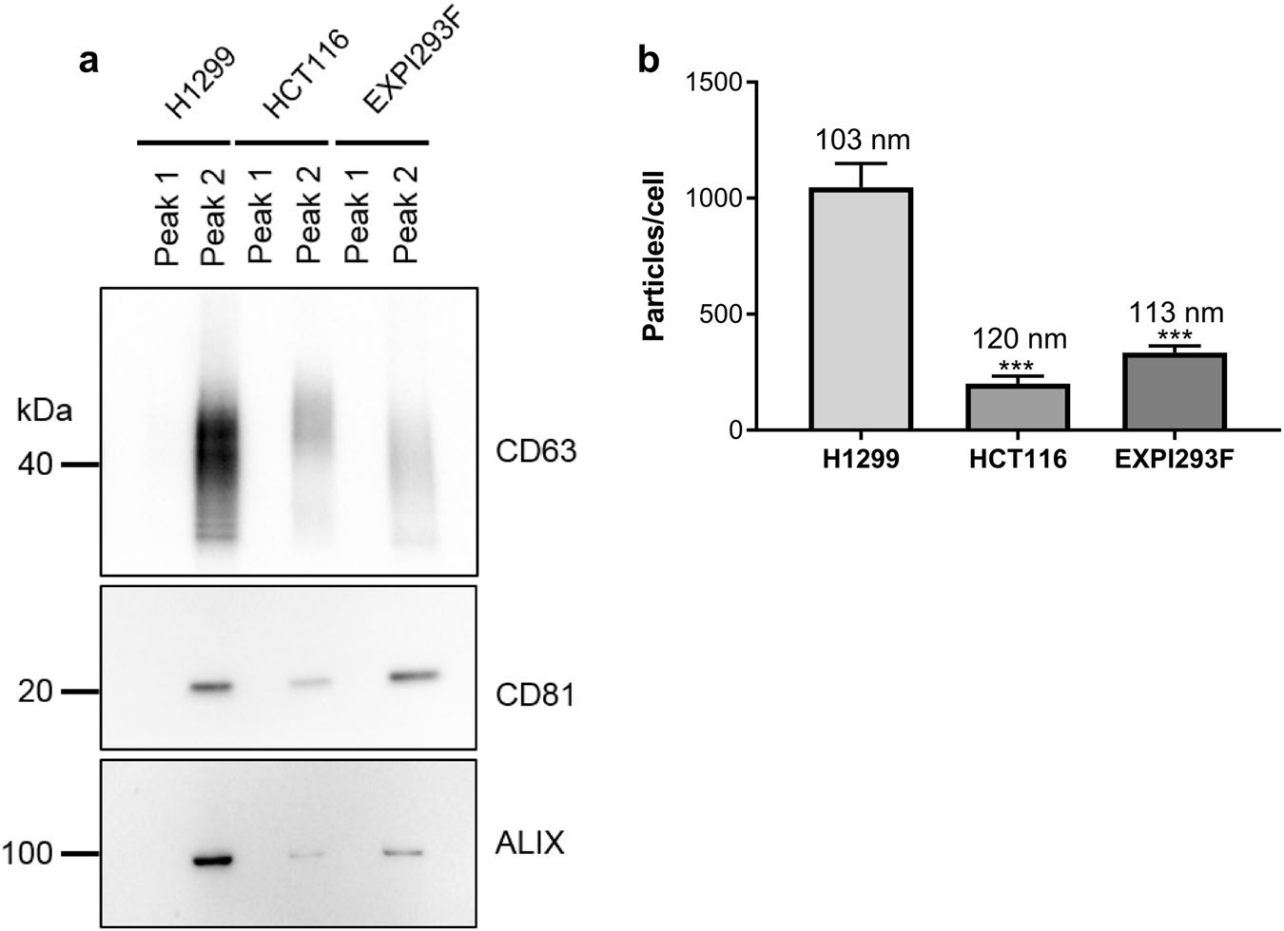
AIEX can be used to isolate EVs from other cell types. EVs were isolated from cancer cell lines H1299 and HCT116 as well as suspension cell line Expi293F™ cells using the optimised AIEX protocol. (**a**) Western blot showed the EV marker content of peak 1 and peak 2 from each cell line n = 3. Full length Western blots can be seen in Supplementary Fig. 10. (**b**) NTA shows the concentration and mean size of EVs in peak 2 isolated from different cell lines. Size is annotated on graph (not significant) n = 3. Error bars ± SEM. Ordinary one way ANOVA, ***p < 0.001.

Finally, we determined whether EVs from different cell line origins can be isolated by AIEX. It has already been shown that mesenchymal stem cell-derived EVs can be isolated by resin based AIEX^31^. As cancer is a dominant topic in the EV field we isolated EVs from H1299 and HCT116 cancer cell lines. Additionally, because scalability is a key point of this study, we used suspension Expi293F™ cells to demonstrate that the technique is also applicable to EVs released by suspension cells. Western blotting showed that from each cell line, peak 2 contained EV markers (Fig. 4a, representative full blots can be seen in figure 10), and NTA showed the presence of particles of EV size in peak 2 (Fig. 4b). A similar number of EVs per cell were isolated from HEK293T, HCT116 and Expi293F™ cells, whereas H1299 cells released on average a minimum of three times more EVs than the other lines. Overall this data confirms that AIEX can indeed be used to isolate and enrich for EVs from different cell types, in a rapid and scalable manner.

## Discussion

In this study, we show that AIEX is a promising alternative technique for the isolation of EVs from different cell lines in an efficient and rapid manner. As summarised in Supplementary Table 1, isolation of EVs using the modified AIEX technique has several advantages. It is a quicker process than UC with less hands-on time necessary, and AIEX isolates EVs of an improved quality and purity to that of TFF and therefore the EVs do not need to undergo a second purification step. Due to the speed, purity and efficiency of the AIEX protocol, it is the single-step EV isolation method that has the most potential to be scaled up for larger cell culture volumes.

Our data confirms that EVs isolated by AIEX are comparable in terms of their size, concentration and morphology to those isolated by ultracentrifugation, which is to date the most commonly used technique in the field for EV isolation. As well as being comparable in size, isolation of EVs by AIEX is also much less time consuming than UC. Isolation from one litre of cell culture supernatant by UC typically takes a full day. On the other hand, for AIEX, 1 litre of cell culture supernatant can be processed in under 3 hours. It is worth noting that cell culture supernatants isolated in this study were subjected to a 0.2 µm filtration step prior to EV isolation, therefore there is potential to increase EV yields even more by removing the filtration step and collecting the larger populations of EVs together. Although 0.2 µm filtration is commonly used in the field for conditioned media clarification^36^, it is important to acknowledge that it is unknown what impact filtration has upon EVs, and whether larger EVs are forced through the filter pores.

TFF isolated EVs were contaminated with proteins and lipid droplets as seen by cryo- electron microscope, suggesting that TFF is not ideal as a single-step large-scale EV isolation procedure if it is desired to exclude contaminating non-vesicular proteins. Indeed, other groups have made use of additional purification steps such as a density cushion for clinical applications^16,20^, which also serves to increase processing times and may give rise to potential losses in yield.

NTA is a very useful technique that uses the ability of particles to scatter light and the particles’ Brownian motion to calculate the size and concentration of particles in solution^37^. Because of the nature of the technique, distinguishing between EVs, aggregated EVs/lipid structures and protein aggregates is not possible. Our NTA data suggests that TFF isolates a larger yield of particles compared to AIEX and UC, however as TFF EVs are heavily contaminated with protein and debris as shown by cryo-electron microscopy, further work would need to be undertaken to decipher if the yield of EVs is in fact larger than AIEX or whether aggregated proteins/lipid droplets are being measured by NTA and contributing to the increased particle number.

To our knowledge, the morphology of EVs isolated by AIEX has not been studied in detail by many groups. Therefore, we analysed the morphology of EVs isolated by AIEX by cryo-electron microscopy. Intact EVs were observed with varying amounts of protein contamination after elution by a linear NaCl gradient; this was improved to show much less contamination when a step gradient was used instead. Upon further optimisation of the AIEX protocol, an increased loading and elution speed was used from 2 ml/minute to 10 ml/minute in combination with a modified step elution. By doing this we could not only reduce the processing time fivefold, but also clearly separate an increased number of protein contaminants away from the EVs. Additionally, the step gradient appeared to isolate vesicles of smaller average size (120 nm versus 179 nm p < 0.05) compared to the linear gradient. Whether this is because the method is more efficient at isolating populations of smaller sized EVs, or because the high salt concentration during elution causes changes in osmotic pressures and consequently alters the lipid dynamics to induce blebbing of vesicles, remains to be elucidated. The short time that the EVs spend in high salt buffer before buffer exchange into PBS, alongside the cryo-electron microscope showing intact EVs of normal morphology suggests that the elution of EVs into high salt is not vastly detrimental. Overall the preparation contained intact EVs with minimal protein contamination and some smaller lipid structures as seen by cryo- electron microscope.

There have been some recent studies that describe the use of AIEX for the isolation of VLPs and EVs. For isolation of VLPs, Steppert *et al*.^30^ use a CIMmultus quaternary amine (QA) strong 1 ml AIEX monolithic column as well as a CIMmultus diethyl amino (DEAE) weak 1 ml column (BIA separations) and saw comparable results using 50 mM HEPES and 350 mM NaCl buffer pH 7.2. They used a 50 mM HEPES and 2 M NaCl buffer to elute VLPs and separate them from contaminating EVs and DNA using both a linear NaCl gradient to 1 M, and a step gradient protocol of 0 mM-760 mM-1100 mM elution buffer^30^. In the step gradient protocol, they observe EVs eluting at 760 mM NaCl and VLPs at 1100 mM NaCl. This is similar to our study where we see EVs eluting at a similar NaCl concentration of 890 mM NaCl and contaminating proteins elute at 335 mM NaCl.

Kim *et al*.^31^ used anion exchange resin (expressQ) rather than a monolithic column to isolate EVs from mesenchymal stem cells using 0.5 M NaCl. A significant improvement in CD63 positive vesicle yield compared to UC at 100,000 g for 1 hour was reported, whereas we observed a modest but not significant improvement of 1.4 times the recovery. This may be due to differences between the studies with Kim *et al*. analysing EVs from mesenchymal stem cells whereas we focussed on the EVs from HEK293T cells, additionally Kim *et al*. use an AIEX resin while we have used an AIEX monolithic column. There has recently been another interesting study whereby DEAE sephadex A50 resin was used to isolate EVs from amniotic fluid by AIEX. The authors found that EVs eluted at 200 mM NaCl were on average smaller (60–100 nm) than EVs eluted by 1 M NaCl (90–220 nm)^38^. This suggests that IEX protocols could potentially be developed that would elute and separate EVs by size. There is also potential to combine AIEX with other chromatography methods depending on the final end-use of the collected EVs, for example it could be combined with affinity purification or size exclusion chromatography to isolate a specific population of EVs. These techniques would not only be useful for the isolation of specific populations of EVs but could also be considered to help in the removal of any remaining contaminating apolipoproteins from EV preparations. To avoid apolipoprotein contamination all together one may choose to use serum free cell culture medium or adapt the cells to grow in the absence of serum where possible.

The current literature suggests that EVs can be isolated by AIEX from numerous cell lines such as mesenchymal stem cells^31^, amniotic fluid^38^ and in our study HEK293T adherent cells, Expi293F™ suspension cells and cancer cell lines H1299 and HCT116. However, one must consider that elution dynamics may be affected in a cell line dependent manner, with the membrane composition of the vesicles released by different cell types altering their surface charge. Therefore, although the optimised method that we have described would serve as a useful starting point for EV isolation with a new cell line, additional modifications may be required.

In conclusion, the final AIEX protocol detailed here shows potential utility for use in both research laboratories as a method for efficient and quick isolation and for use in a clinical setting where larger volumes of culture supernatant need to be processed in a controlled environment. In addition, the observation that a single pass through a 1 ml monolithic AIEX column can isolate 2.4*10^11^ of the EVs present in 1 litre of conditioned media in fewer than 3 hours confirms this notion. This technique is therefore a step forward in terms of speed and scalability of EV isolation and an approach that we feel can bring significant benefits to the continued future clinical exploitation of EVs.

## Materials and Methods

### Cell culture

HEK293T cells (ATCC) were cultured at 37 °C and 5% CO_2_ in high glucose DMEM supplemented with 10% FBS and 2 mM L-glutamine (all Gibco). H1299 cells (ATCC) were cultured under the same conditions using RPMI media (Gibco) and HCT116 cells (ATCC) in McCoys 5 A media (Gibco). Expi293F™ cells (Thermo Fisher Scientific) were cultured in synthetic Expi293 expression media™ (Thermo Fisher Scientific) at 37 °C at 125 rpm in 2 litre Corning® roller bottles (Sigma).

For EV collections, adherent cell culture media was supplemented with 10% exosome depleted serum (Thermo Fisher Scientific) 48 hours prior to collection. For Expi293F™ cells no supplementation was necessary. For EV collection technique comparison experiments HEK293T cells were plated in Nunc™ triple layer flasks for 24 hours (Thermo Fisher Scientific), washed twice with PBS and supplemented with 70 ml DMEM with 10% exosome depleted serum. Conditioned media was harvested from 3 triple layer flasks (210 ml total volume), from an average of 6.4*10^8^ cells, for each EV collection technique. In AIEX optimisation experiments, for optimal experimental speed, scalability and ease of cell culture handling, cells were plated into Corning® 10 layer CellSTACKs® (Sigma) into 1 litre of DMEM containing 10% exosome depleted serum. Cells conditioned media for 48 hours before EVs were harvested from an average of 7.8*10^8^ cells.

### Clarification of conditioned media

Conditioned media was spun at 300 g for 10 minutes and filtered using a volume appropriate (250 ml or 1000 ml) 0.2 µm aPES vacuum bottle top filter (Thermo Fisher Scientific). For media-only control conditions, the media that had not been exposed to cells was subjected to a 0.2 µm filtration.

### UC

Clarified conditioned media was transferred into Beckman polyallomer 38 ml ultracentrifuge tubes and spun at 100,000 g in an SW32 Ti rotor for 70 minutes. Supernatant was removed and pellets re-suspended in PBS (Sigma) and pooled. EV samples were washed in 38 ml PBS and re-pelleted using a 100,000 g spin. Pellets were re-suspended in 100–200 µl PBS or TBS and frozen (−20 °C) in aliquots.

### TFF

The method was adapted from Lamparski *et al*.^20^. Clarified conditioned media was processed using an ÄKTA flux S TFF system (GE healthcare) and passed through a 500 kDa cut off hollow fibre membrane (UFP-500-C-4A GE healthcare) at 28 ml/min with no back pressure applied. The transmembrane pressure did not exceed 0.3 psi. The flow-through (<500 kDa) was discarded and the clarified media circulated until it had reduced in volume to 20 ml. The sample was diafiltrated with 100 ml PBS four times. The retentate (20 ml) was harvested and 20 ml PBS was used to wash the membrane before also being harvested. The 20 ml retentate plus 20 ml wash sample were combined and concentrated using centrifugal concentrators (100 kDa membranes Merk-millipore) to 100–200 µl in PBS. Samples were aliquoted and frozen (−20 °C).

### AIEX

A 1 ml CiMmultus™ QA (6 µm) anion exchange column (BIA Separations) was equilibrated using buffer A (50 mM HEPES, 100 mM NaCl). Clarified conditioned media was applied to the column at 2 ml/minute using an ÄKTA Pure chromatography system (GE Healthcare). Following sample loading, the column was washed with Buffer A for 20 column volumes (CV). A linear gradient of 0–50% Buffer B (50 mM HEPES, 2 M NaCl) was used to elute EVs from the column at 2 ml/minute over 20 CVs with 1.5 ml fractions being collected. The column was washed with 100% Buffer B for 5 CV and re-equilibrated with 5 CVs of Buffer A. A 100 µl sample was taken from each fraction corresponding to the UV absorbance peak (280 nm), and the remaining 900 µl of each of the fractions were pooled and concentrated using 100 kDa centrifugal concentrators to1 ml. 13 ml of PBS or TBS was used to dilute and buffer-exchange the concentrated EV sample and this was re-concentrated to 1 ml. This step was repeated twice to ensure thorough buffer exchange. The sample was concentrated to its final 100 µl volume and aliquoted and frozen (−20 °C). In the optimised AIEX protocol, the flow rate of sample application and elution was increased to 10 ml/minute. A step elution of 10 CV at 10% NaCl (335 mM) and 10 CV at 40% NaCl (890 mM) was used. The fractions from each of the two resultant peaks were then pooled separately, buffer exchanged and concentrated to 200 µl as described above.

### SDS-Page electrophoresis

Cells were lysed with 50 mM Tris/1% SDS lysis buffer, homogenised with a 23 G needle and debris pelleted by centrifugation for 10 minutes at 13,000 rpm. 40 µg of cell lysate, or 7.5 µl of EV sample in 4 times LDS loading buffer (Thermo) plus 10% β-mercaptoethanol reducing agent (Sigma), were loaded and ran on 15 well 4–12% Bis-Tris gradient gels (NuPage Thermo) at 180 mV for 50 minutes using MOPs running buffer (50 mM Tris, 50 mM MOPS, 0.1% SDS, 1 mM EDTA). Non-reducing conditions were used for detection of CD63. SeeBlue™ Plus2 and MagicMark™ (Thermo Fisher Scientific) protein standards were used.

### Western blotting

Gels were transferred using the iBlot system according to manufacturer’s instructions (Thermo Fisher Scientific). Membranes were blocked in appropriate blocking buffer. Primary antibody was applied to membranes overnight at 4 °C with agitation. Membranes were washed 3 times for 10 minutes in TBS-T (100 mM Tris-HCl, 150 mM NaCl & 0.5% Tween). Secondary anti-mouse/rabbit/goat HRP conjugated antibody was applied to membranes for 45 minutes at room temperature and washed as above. Blots were developed using equal volumes of ECL extreme developing reagent (Expedion) and Biorad ChemiDoc imaging system. Images were acquired and saved using Image Lab 5.0. For antibodies, blocking solutions and dilutions see Supplementary Table 2.

### Nanoparticle tracking analysis

Biological replicate samples were collected independently and stored at −20 °C before being measured on the LM10 NanoSight (Malvern Instruments) equipped with a sample chamber and a 405 nm laser. The software used for data capture and analysis was NTA 3.2 Dev Build 3.2.16. EVs were diluted in PBS and injected into the sample chamber using a 1 ml syringe until the chamber was filled. Per sample, three 30 second measurements were performed at camera level 14 or 15 at room temperature. A script was used which allowed the sample to be advanced manually between each measurement. Between each measurement the chamber was washed with PBS until no particles were detected in the field of view. Particle size distributions shown in Fig. 2b–d are based on minimum of 1500 particles to 5400 particles.

### Protein concentration determination

Protein concentration of EV preparations and lysates was determined using 2 µl of sample with a Nanodrop spectrophotometer (Thermo Fisher Scientific) with PBS or lysis buffer respectively as a blanking solution.

### Cryo-electron microscopy

EV samples were applied to glow-discharged (1 minute in 0.2 mbar air) 300 mesh Quantifoil holey carbon EM grids and were vitrified using a Vitrobot Mark IV (FEI Company, The Netherlands) at 4 °C and 100% humidity. Excess sample was removed by blotting once for 1 second with filter paper. The blotted grids were plunged into liquid ethane. Cryo- electron microscope imaging was performed on a Titan Krios electron microscope (FEI Company, The Netherlands) operated at 300 kV. Images were recorded on a Falcon II camera (FEI Company, The Netherlands). Images were recorded with a pixel size of 0.45 nm at 5 µm defocus.

### Peptide mapping

Three biological replicates of AIEX EVs isolated from peak 1 and peak 2, were analysed in triplicate for label free LC-MS/MS. EVs were lysed in 6 M urea. Protein was precipitated using acetone, digested using 1 µg trypsin, reduced using 100 mM DTT and alkylated using 150 mM iodoacetamide followed by an overnight digest with 1 µg of trypsin, all of the above reactions were in 50 mM ammonium bicarbonate, pH 8.0. Samples were acidified using 0.1% formic acid/5% acetonitrile and cleaned up using Pierce C18 spin columns following manufacturer’s instructions. Peptides were eluted with 0.1% formic acid/5% acetonitrile, dried and reconstituted in 0.1% formic acid. Yeast alcohol dehydrogenase tryptic digest was spiked in to allow for quantification. 4 µl of each sample (normalised to 0.8 µg protein/µl) was run in triplicate on a NanoAcquity LC coupled to a Synapt G2 mass spectrometer (Waters). A 90-minute reversed phase gradient was run for each sample and ion mobility MS^E^ data was collected. Data was analysed using Waters Prognesis QI for Proteomic software searching against the Human Uniprot database.

### Data analysis

All data handling was performed in Excel and any analysis and statistics were calculated using Graph Pad Prism 7. For normally distributed data, an unpaired t-test was used for data sets with two conditions, or one-way ANOVA with multiple comparisons was used for data sets with multiple groups. P values of less than 0.05 were considered to be statistically significant. *p < 0.05. **p < 0.01. ***p < 0.001. ****p < 0.0001. Cryo-electron microscope images were viewed and edited using ImageJ software (National Institutes of Health. Figures were made using Inkscape.

### Data availability statement

Data sets generated during this study are available from the corresponding author upon reasonable request.

## Electronic supplementary material

Supplementary information

Supplementary Dataset 1

Supplementary Dataset 2
