# Supplementary material for: Rapid isolation and enrichment of extracellular vesicle preparations using anion exchange chromatography
Source: Sci Rep. 2018 Apr 10;8:5730. doi: 10.1038/s41598-018-24163-y (PMC5893571; doi:10.1038/s41598-018-24163-y)
Supplement: Supplementary file 1 — Supplementary information [file 41598_2018_24163_MOESM1_ESM.pdf]

Rapid isolation and enrichment of extracellular vesicle preparations using anion exchange chromatography

Nikki Heath[1*], Lois Grant[1], Taiana Maia De Oliveira[2], Rachel Rowlinson[1], Xabier Osteikoetxea[1], Niek Dekker[3] & Ross Overman[1]

[1] Discovery Sciences, IMED Biotech Unit, AstraZeneca, Alderley Park, UK
[2] Discovery Sciences, IMED Biotech Unit, AstraZeneca, Cambridge, UK
[3] Discovery Sciences, IMED Biotech Unit, AstraZeneca, Gothenburg, Sweden

[*]Address for correspondence: nikki.heath@astrazeneca.com

Supplementary data and files

Supplementary files.

Supplementary file 1: Mass spectrometry Peptide mapping data of the significantly enriched proteins found in peak 1 and peak 2. To give the reader a broader view of the peptide mapping data, all proteins significantly increased in peak 1 and peak 2 are shown in the excel file.

Supplementary file 2: Mass spectrometry peptide mapping data of top 460 proteins identified in peak 1 and peak 2 in order of abundance (fmol). This spreadsheet details the peptide mapping data of peak 1 and peak 2 independently of one another and without statistical analysis. All runs for each condition (3 technical repeats for each of the 3 biological repeats) was averaged and the data is presented in order of abundance from highest to lowest fmol column load.

Supplementary figure 1

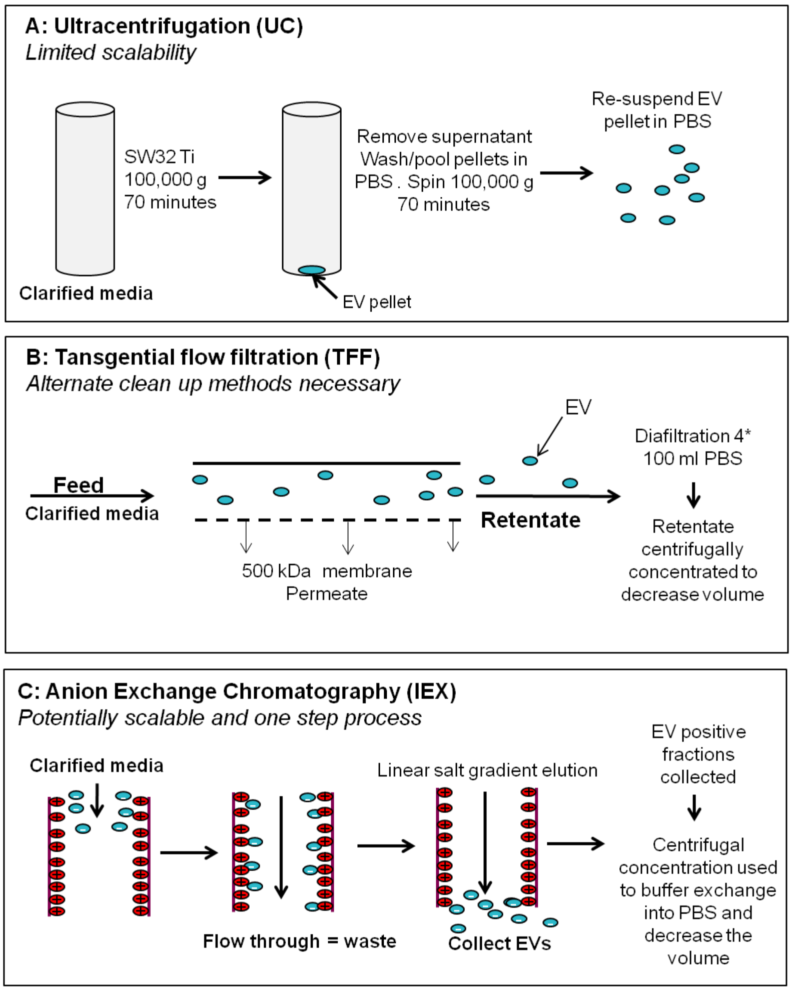

Supplementary figure 1: Isolation of extracellular vesicles by UC, TFF and AIEX. Schematic diagram depicting how EVs are isolated by UC (A), TFF (B) and AIEX (C) techniques. Conditioned media was collected from HEK293T cells and clarified using a 300 g spin and a 0.2 µm aPES filter. EVs were isolated from clarified conditioned media by ultracentrifugation at 100,000 g for 70 minutes. Resultant pellets were pooled and re-pelleted by a further centrifugation. Alternatively, TFF was used to filter and concentrate conditioned media through a 500 kDa cut off hollow fibre cartridge. Anything smaller than 500 kDa passed through the membrane as permeate to waste, anything bigger was retained (including EVs). Once concentrated the residual volume was diafiltrated with 100 ml PBS 4 times before collecting a final volume of 20 ml, and concentrating further using centrifugal concentration. For AIEX the clarified conditioned media was applied to an anion exchange monolithic column and positively charged particles bound. Particles were eluted using a linear NaCl gradient up to 1 M. EV positive fractions were pooled, buffer exchanged and concentrated. EV preparations isolated by each technique were isolated from the same cell number and re-suspended in the same final volume of PBS or TBS for further comparative analysis.

Supplementary figure 2

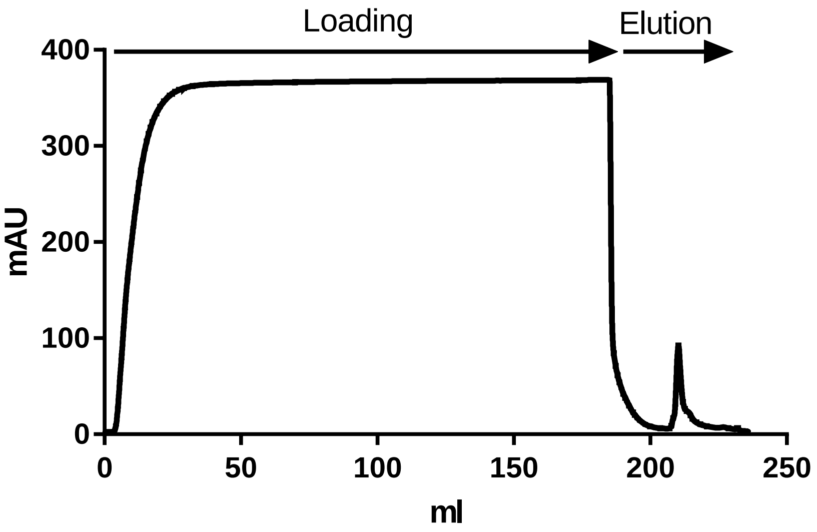

Supplementary figure 2: AIEX chromatogram showing loading of clarified conditioned media and elution of EVs. Clarified conditioned media from HEK293T cells was applied to the AIEX 1 ml column at 2 ml/minute and eluted using a linear NaCl gradient from 100 – 765 mM. Chromatogram representative of the A280 absorbance (mAu) measured during the loading and elution.

Supplementary figure 3

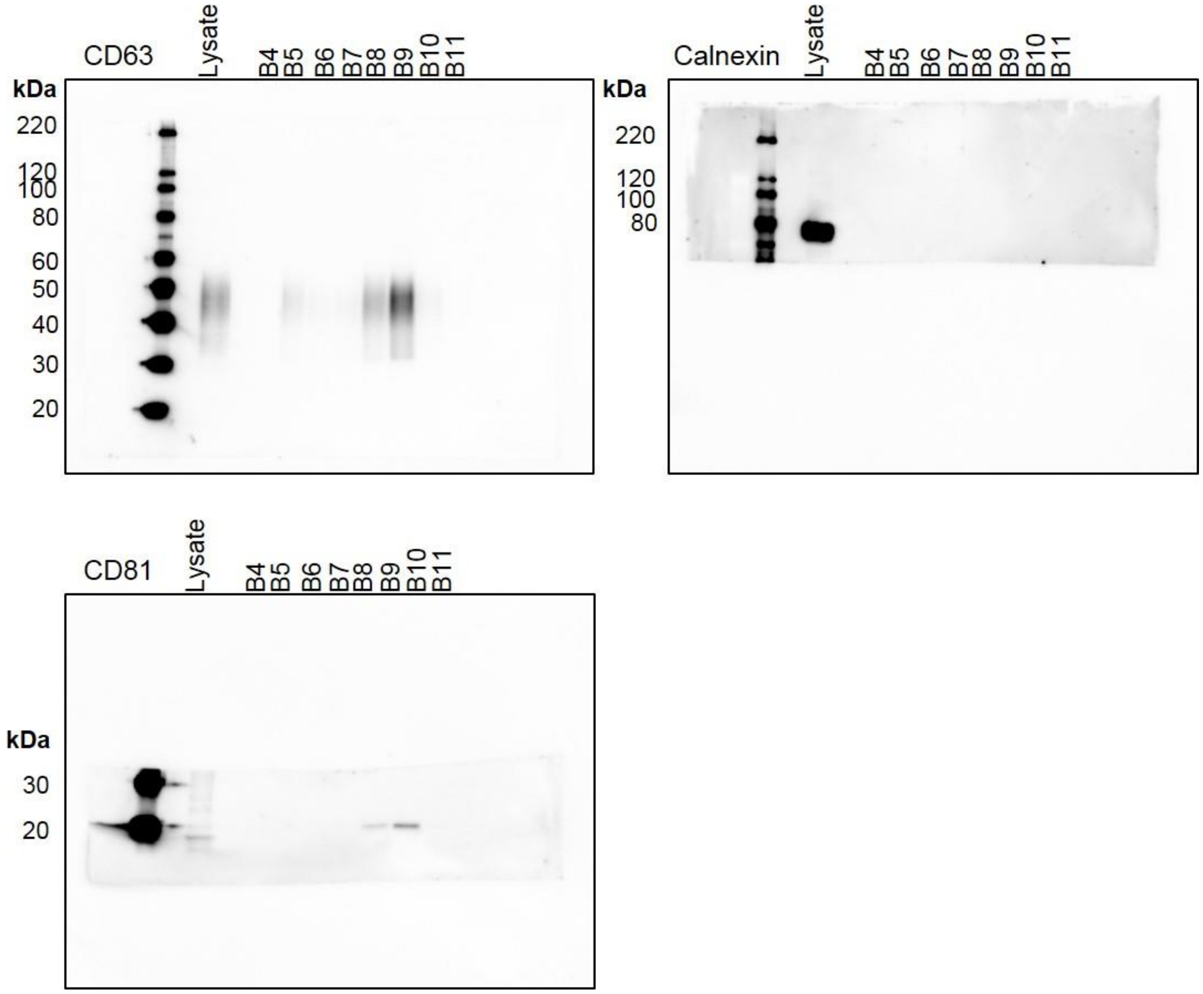

Supplementary figure 3: Full length Western blots of EVs isolated by AIEX. Whole Western blots for data shown in figure 1.

Supplementary figure 4

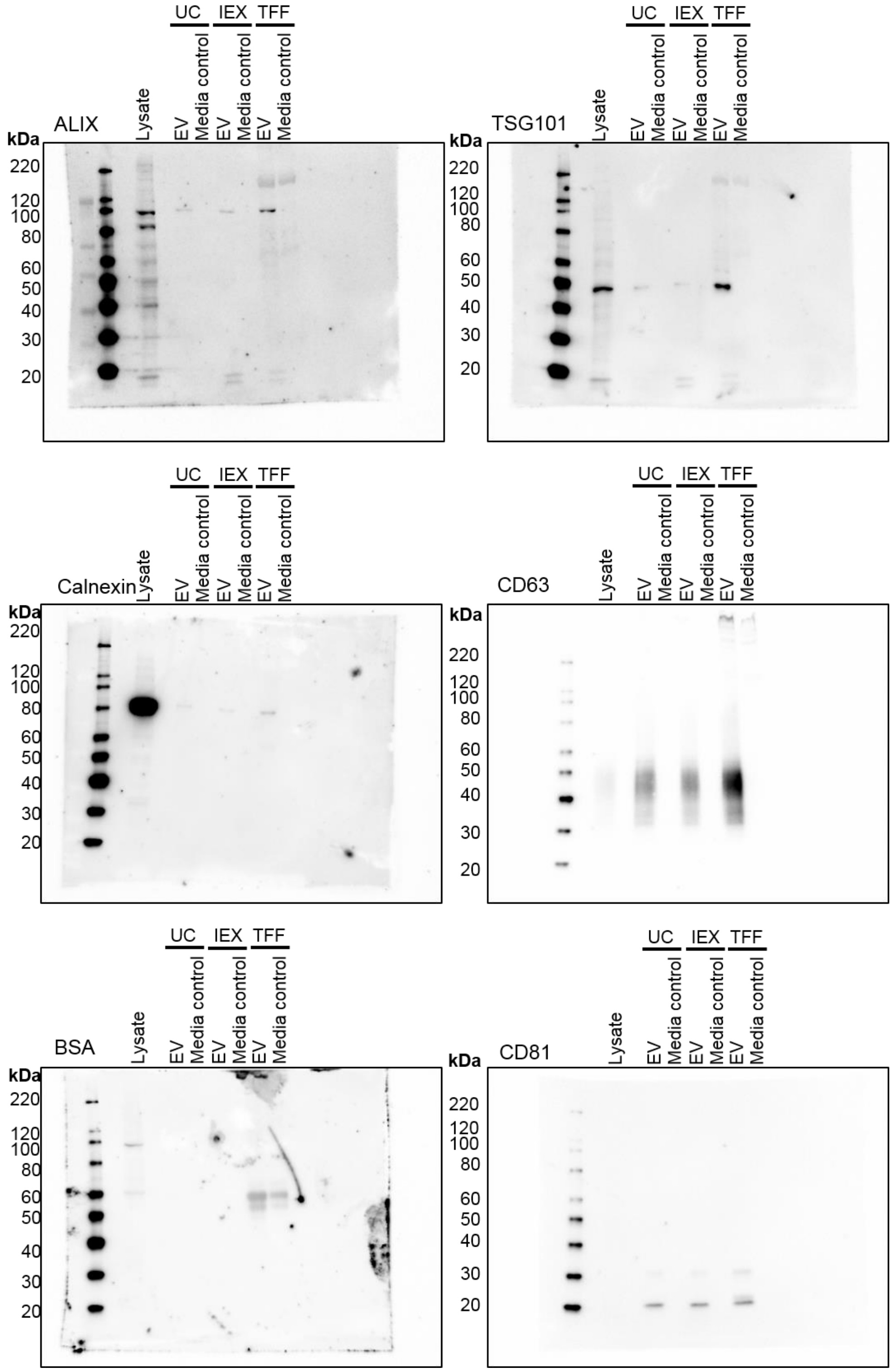

Supplementary figure 4: Full length Western blots of EVs isolated by AIEX, UC and TFF. Full length Western blots for data shown in figure 2.

Supplementary figure 5

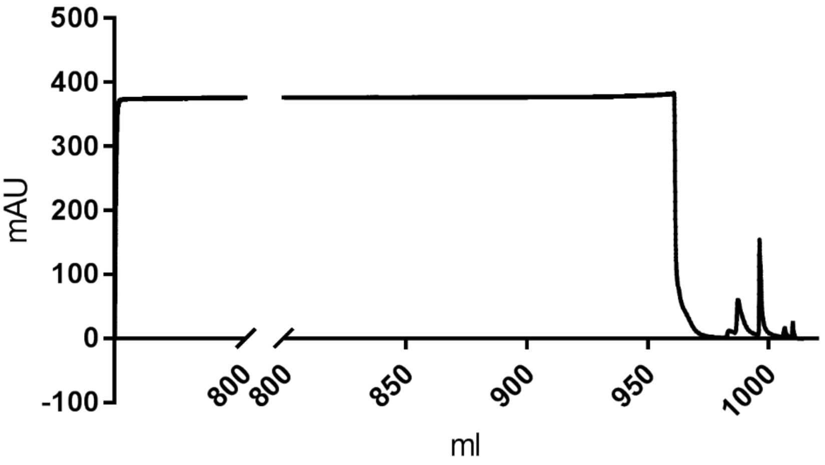

Supplementary figure 5: Representative image of full chromatogram upon optimisation of AIEX protocol. Clarified conditioned media from HEK293T cells was applied to the AIEX 1ml column at 10 ml/minute and eluted using a step gradient of 335 mM NaCl and 890 mM NaCl resulting in two defined peaks. Chromatogram representative of the A280 absorbance (mAu) measured during the loading and elution.

Supplementary figure 6

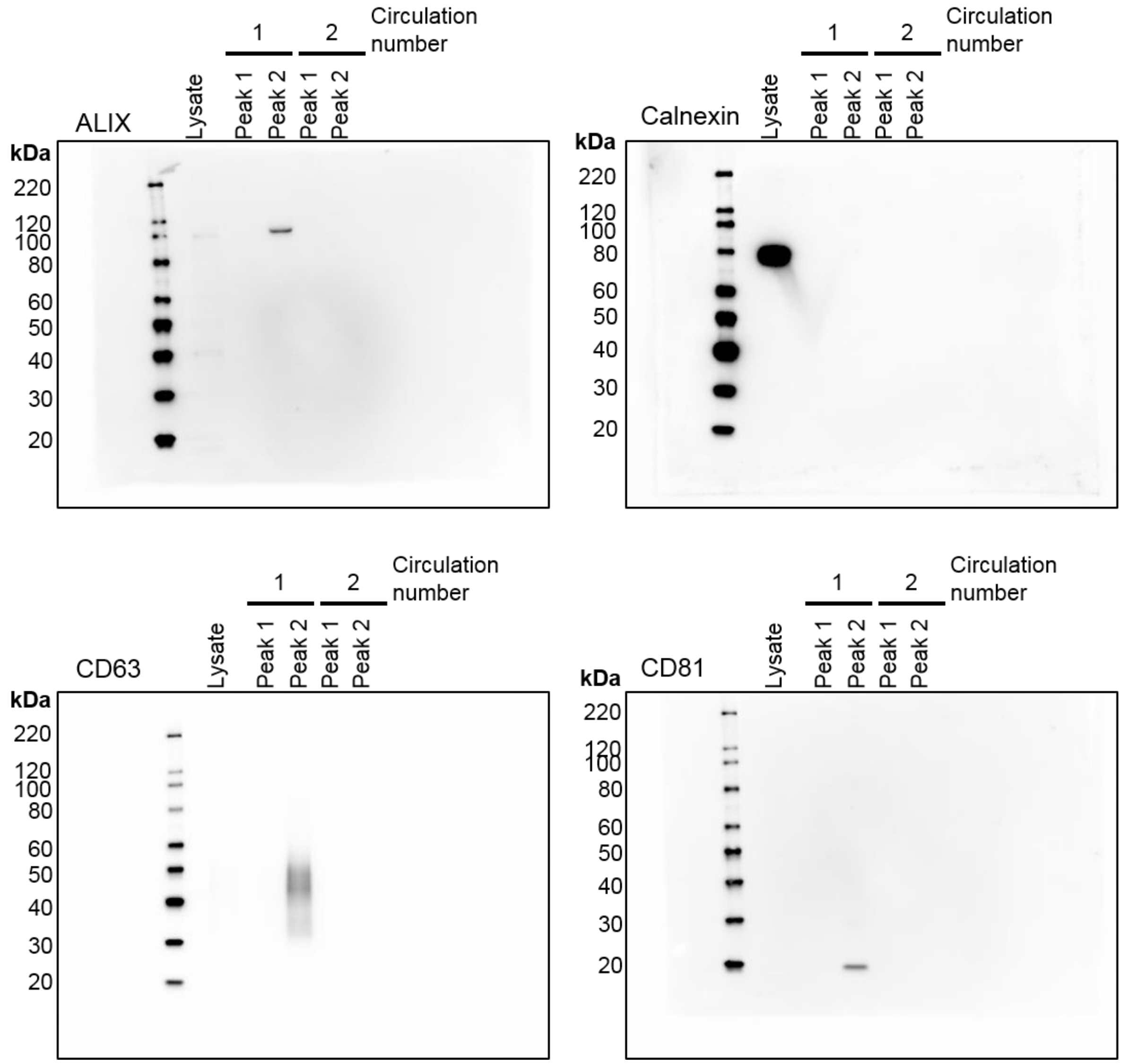

Supplementary figure 6: Full length blots of EVs isolated by optimised AIEX protocol. Full length Western blots of data shown in figure 3.

Supplementary figure 7

A

Peak 1

Pathway name	Number of genes
Ribosome	46
Gap Junction	7
Systemic lupus erythematosus	8
Toxoplasmosis	7
Pathogenic E. coli infection	5

B

Peak 2

Pathway name	Number of genes
Endocytosis	23
Neurotrophin signalling pathway	12
Pancreatic secretion	11
Leukocyte transendothelial migration	11
Pathogenic E. coli infection	9

C

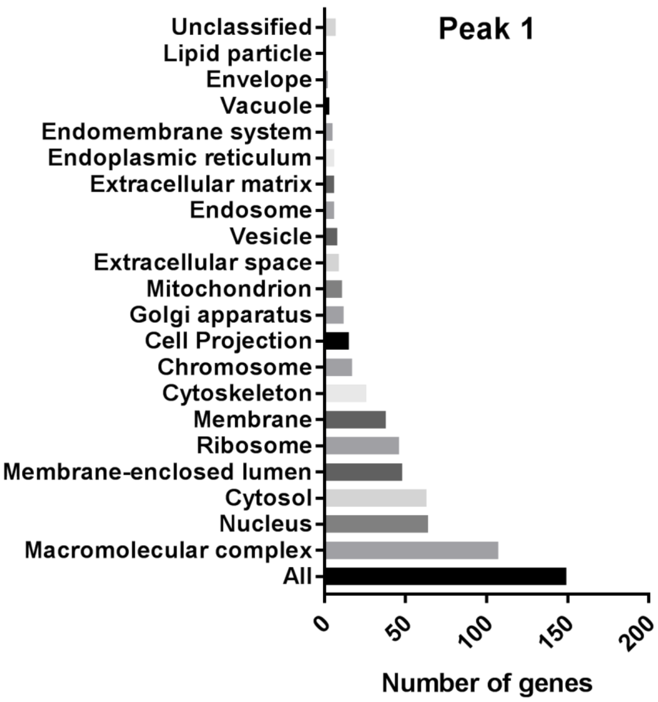

D

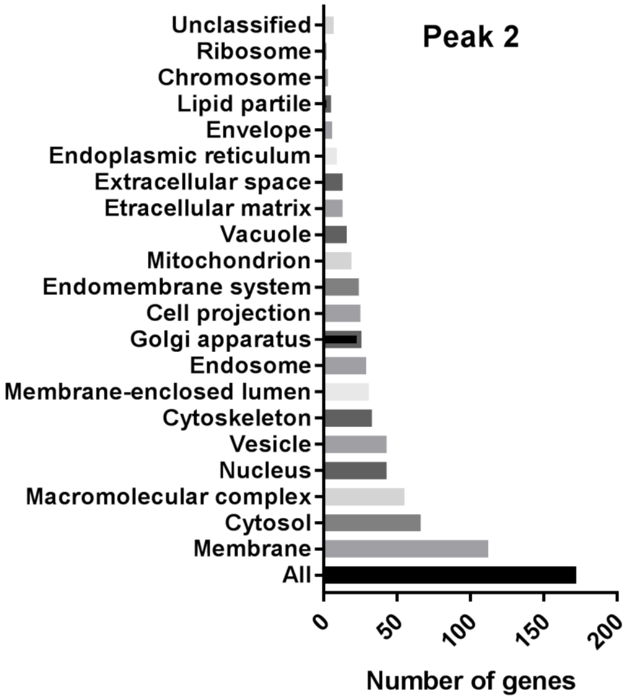

Supplementary figure 7: Peptide mapping of EVs derived from Peak 1 and Peak 2. Analysis of peptides significantly (p equal to or less than 0.05) enriched in peak 1 and peak 2 was carried out using web based gene set analysis toolkit (WebGestalt)[1]. Data presented are the top five pathways in which proteins that are enriched in peak 1/2 are involved (A/C) and the cellular compartment in which the proteins from peak 1/2 are most commonly found (B/D).

Supplementary figure 8

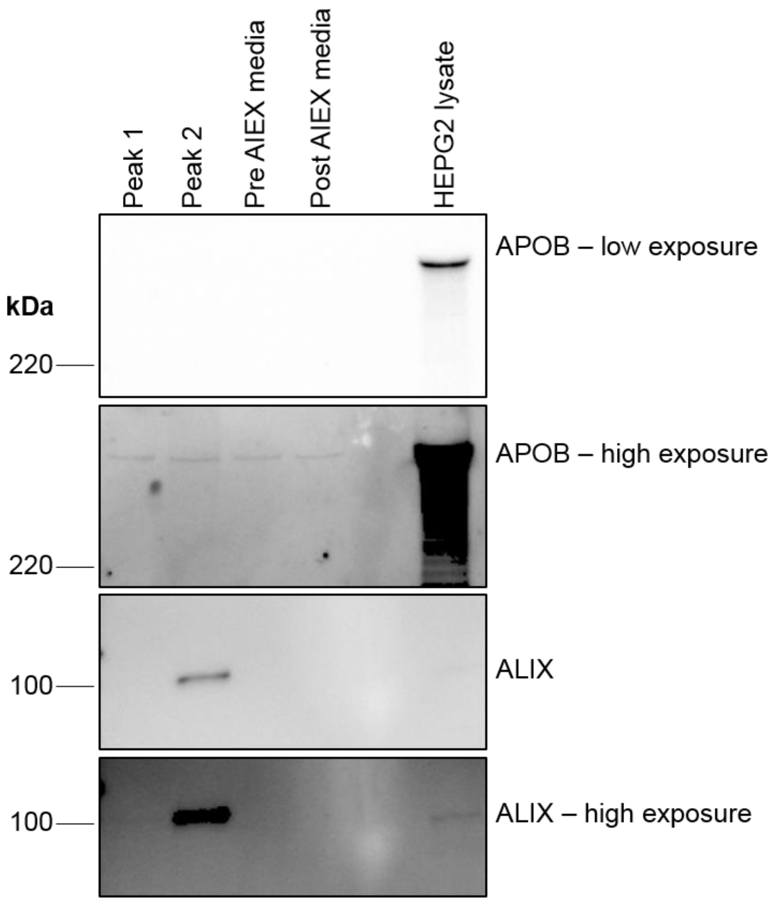

Supplementary figure 8: Western blot of apolipoprotein ApoB. EVs were collected by AIEX from 1 litre of HEK293T conditioned media and resuspended in 100 µl PBS. 7.5 µl peak 1, peak 2, un-concentrated conditioned media before AIEX and conditioned media from post AIEX were ran on a 10 well 3 – 8 % Tris-acetate gradient gel (NuPAGE, Thermo Fisher Scientific) and Western blotted for ApoB and ALIX. HEPG2 cell lysates were used as a positive control for ApoB. ApoB antibody has cross-reactivity with human and bovine sequences as stated on manufacturers website. Antibody details can be found in supplementary table 2. n=5.

Supplementary figure 9

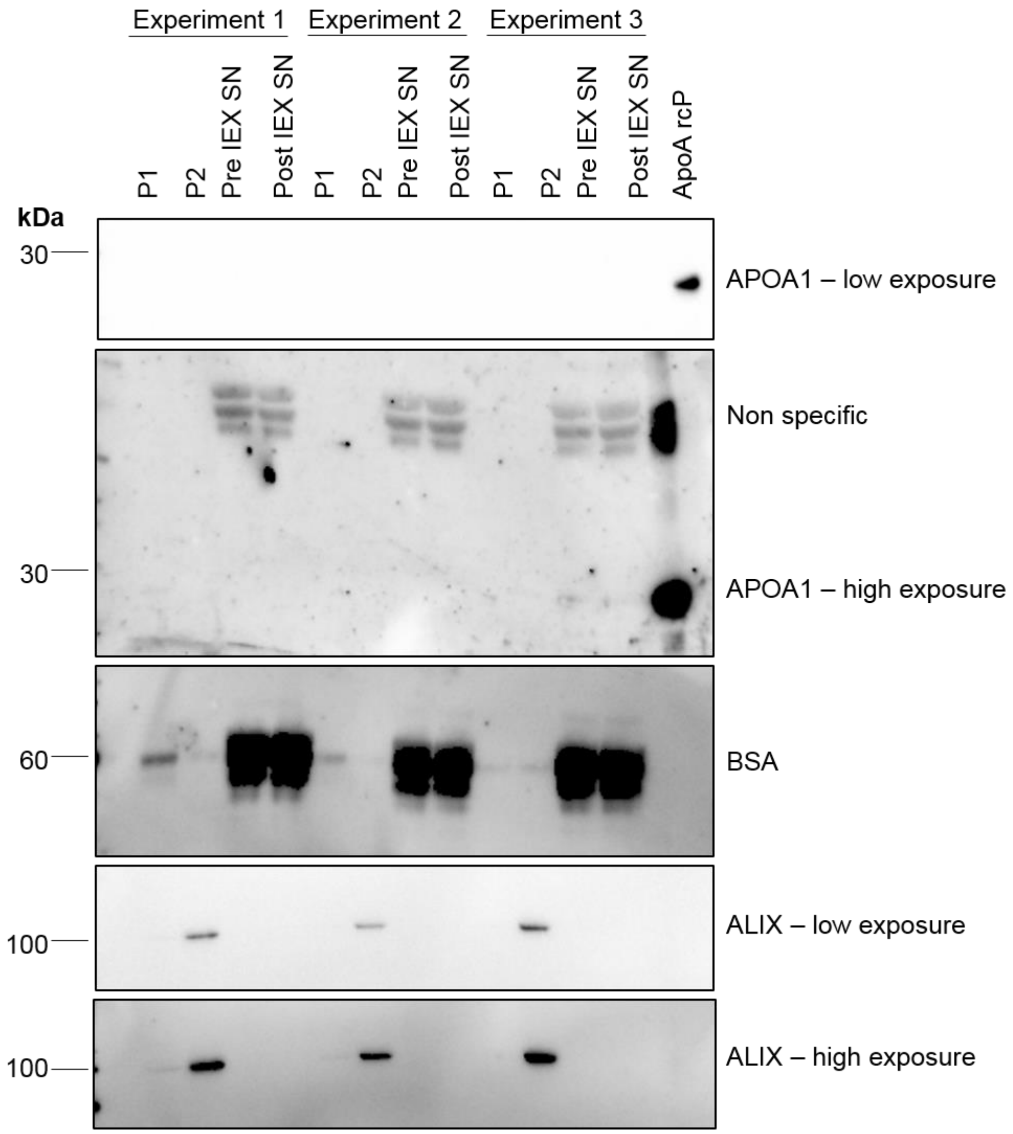

Supplementary figure 9: ApoA1 and BSA Western blots. 7.5 µl peak 1, peak 2, un-concentrated conditioned media and AIEX conditioned media flow-through were ran on 4 – 12 % Bis tris gels (NuPAGE, Thermo Fisher Scientific). Peak 1 and 2 were in a volume of 100 µl, conditioned media pre- and post-IEX was in a total volume of 1000 ml. Western blots were probed for ApoA1, BSA and ALIX. Human ApoA1 recombinant protein (Abcam, ab50239) was used as a positive control for ApoA1. ApoA1 antibody as stated by Thermo Fisher Scientific has cross reactivity with human and bovine forms of the protein. n=3, all three experiments shown.

Supplementary figure 10

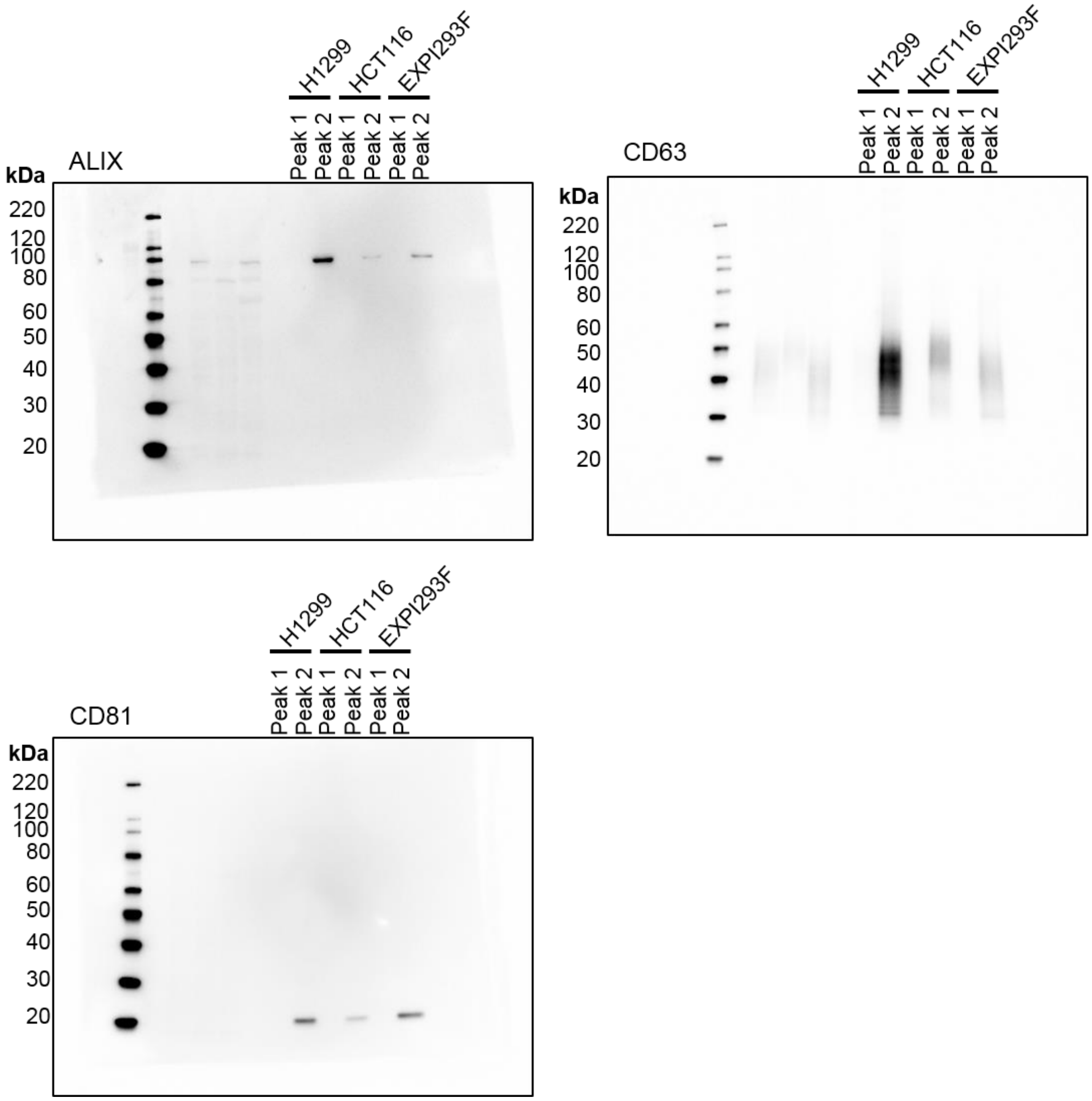

Supplementary figure 10: Full length Western blots of EVs isolated by AIEX from H1299, HCT116 and EXPI293F cell lines. Full length Western blots of data shown in figure 4.

Supplementary table 1: Table showing the advantages and disadvantages of isolating EVs by AIEX, UC and TFF. Here, the time taken, scalability, yield and purity of EV preparations as isolated by a single step protocol by UC, AIEX or TFF are considered and summarised. * = low. **** = high. Overall UC has good yield and purity and no further purification steps are necessary, however the isolation protocol is long and there is little room for scalability. TFF has potential for scalability and high yields however preparations are highly contaminated and need further purification and concentration steps. AIEX with a final EV concentration step enriches for EVs but not for contaminating proteins such as BSA, Apo1 and ApoB, and the technique has a huge amount of potential for scalability.

	**UC**	**AIEX**	**TFF**
**Time (for 1 litre)**	7 hours	**3 hours**	3 hours
**Potential for scalability**	*	********	****
**Protein contamination**	**	******	****
**Particle yield**	*	******	****
**Final EV concentration step necessary?**	No	**Yes**	Yes
**Additional purification necessary?**	No	**No**	Yes

***Low**
******High**

Supplementary table 2: The antibodies used in the study company and usage information. Information of all antibodies used in this study is provided, including company, product number, species, the blocking agent used and the dilution in which it was used.

Antibody/ species	Company	Dilution	Blocking agent
CD63/ Mouse	Abcam (ab59479)	1:1000	5 % Milk
CD81/ Mouse	Santa Cruz biotechnology (sc-166029)	1:300	3 % BSA
ALIX/ Mouse	Abcam (ab88743)	1:1000	3 % BSA
TSG101/ Mouse	Abcam (ab38)	1:1000	3 % BSA
B-actin/ Mouse	Thermo Fisher Scientific (BA3R)	1:1000	5 % Milk
Calnexin/ Rabbit	Abcam (ab22595)	1:1000	5 % Milk
BSA/ Rabbit	Thermo Fisher Scientific (A11133)	1:1000	5 % Milk
APOB/ Goat	Abcam (ab7616)	1:1000	5 % Milk
APOA1/ Mouse	Thermo Fisher Scientific (MA1-83002)	1:1000	5 % Milk
Anti-mouse-HRP	Sigma Aldrich (A9044)	1:10 000	5 % Milk/3 % BSA
Anti-rabbit-HRP	Sigma Aldrich (A0545)	1:10 000	5 % Milk/ 3 % BSA
Anti-Goat-HRP	Sigma Aldrich (A8919)	1:10 000	1 % Milk / 1 % BSA

References

1 Wang, J., Duncan, D., Shi, Z. & Zhang, B. WEB-based GEne SeT AnaLysis Toolkit (WebGestalt): update 2013. *Nucleic Acids Res* **41**, W77-83, doi:10.1093/nar/gkt439 (2013).
